# DNA polymerase ι is acetylated in response to S_N_2 alkylating agents

**DOI:** 10.1038/s41598-019-41249-3

**Published:** 2019-03-18

**Authors:** Justyna McIntyre, Aleksandra Sobolewska, Mikolaj Fedorowicz, Mary P. McLenigan, Matylda Macias, Roger Woodgate, Ewa Sledziewska-Gojska

**Affiliations:** 1 Institute of Biochemistry and Biophysics, Polish Academy of Sciences, ul. Pawinskiego 5a, 02-106 Warsaw, Poland; 2 Laboratory of Genomic Integrity, National Institute of Child Health and Human Development, National Institutes of Health, Bethesda, MD 20892-3371 USA; 3 Laboratory of Molecular and Cellular Neurobiology, International Institute of Molecular and Cell Biology, ul. Ks. Trojdena 4, 02-109 Warsaw, Poland

## Abstract

DNA polymerase iota (Polι) belongs to the Y-family of DNA polymerases that are involved in DNA damage tolerance through their role in translesion DNA synthesis. Like all other Y-family polymerases, Polι interacts with proliferating cell nuclear antigen (PCNA), Rev1, ubiquitin and ubiquitinated-PCNA and is also ubiquitinated itself. Here, we report that Polι also interacts with the p300 acetyltransferase and is acetylated. The primary acetylation site is K550, located in the Rev1-interacting region. However, K550 amino acid substitutions have no effect on Polι’s ability to interact with Rev1. Interestingly, we find that acetylation of Polι significantly and specifically increases in response to S_N_2 alkylating agents and to a lower extent to S_N_1 alkylating and oxidative agents. As we have not observed acetylation of Polι’s closest paralogue, DNA polymerase eta (Polη), with which Polι shares many functional similarities, we believe that this modification might exclusively regulate yet to be determined, and separate function(s) of Polι.

## Introduction

The transfer of an acetyl group from acetyl coenzyme A to a specific site on a protein is one of the major posttranslational modifications and one of many that modify lysine residues^1,2^. Lysine acetylation was first discovered in histones and its significance in transcription control has been demonstrated^3,4^. Besides transcription, acetylation regulates many other cellular processes including the cell cycle, proliferation, apoptosis, DNA recombination, stress response and DNA repair^5–7^. Lysine acetylation can have a profound and diverse impact on modified proteins as it can influence protein stability, localization, enzymatic activity, as well as DNA and protein binding^8–11^. Acetylation is a dynamic modification catalyzed by acetyltransferases that can be reversed by deacetylases. The lysine acetyltransferases (KATs) are grouped in three major families and one of them, p300/CREB-binding protein (CBP), consists of just two members, p300 and CBP^12^.

Mammalian p300 and CBP are paralogs sharing 86% amino acid identity in their aminotransferase domain and while they are conserved in metazoans, they do not have detectable sequence homology with other KATs^12,13^. The enzymes possess a bromodomain that recognizes acetylated substrates and multiple other non-catalytic domains involved in protein binding^14^. Additionally, a non-canonical, but functional RING domain, connects the enzymes with ubiquitination processes^15,16^. p300/CBP interact with over 400 proteins and act as network hubs in different cellular pathways, often in complexes controlling transcriptional activation^17^. Defects in these acetyltransferases have been linked to human diseases, including several types of cancer, heart malfunction, diabetes mellitus, as well as Rubinstein-Taybi syndrome, which is characterized by developmental abnormalities and cancer predisposition^17,18^. On the other hand, due to the fact that p300/CBP are involved in the regulation of many tumor-relevant proteins including p53, c-myc, or BRCA1, many therapeutic strategies targeting p300/CBP are under investigation [reviewed in^19–22^]. Despite the high homology between p300 and CBP, there is accumulating evidence to suggest that CBP and p300 are not fully redundant but, due to differential association with other proteins, or diversity in their substrate specificity, also have unique roles *in vivo* [reviewed in^18^].

p300/CBP-directed lysine acetylation seems to play an important and diverse role in DNA replication and the DNA damage response. The p300/CBP-acetylated proteins are engaged in DNA damage recognition, signaling and most DNA repair pathways [^23,24^, reviewed in^5^]. However, until recently, there is little indication for the involvement of p300/CBP in the regulation of DNA damage tolerance mechanisms. Our previous report was the first to suggest such a possibility^25^. We proposed that p300 acetyltransferase inhibition influences polyubiquitination of DNA polymerase iota (Polι), a non-canonical polymerase involved in DNA translesion synthesis (TLS).

Even though TLS and other DNA damage tolerance processes do not actually repair DNA lesions, they play a crucial role in cell survival and genomic stability maintenance [reviewed in^26^]. They are particularly important in dividing cells, as DNA synthesis by replicative polymerases is stalled by lesions in the template DNA. The threat of a replication block may be circumvented by employing TLS polymerases, which are able to incorporate a nucleotide opposite the normally replication-blocking lesion^27^. Depending on the lesion and the TLS polymerase employed, TLS can be accurate, or highly error-prone^28^.

DNA polymerases (Pols) eta (η), iota (ι), kappa (κ) and Rev1 belong to the Y-family of DNA polymerases that are best known for their TLS activity^29^, which is credited to their flexible and capacious active site, thereby allowing them to accommodate damaged nucleotides. Polη, a flagship member of the Y-family DNA polymerases, is able to correctly bypass a thymine-thymine cyclobutane pyrimidine dimer (CPD), which is the most common mutagenic UV-light induced DNA lesion. Accordingly, the lack of Polη in XPV cells makes them sensitive to UV-radiation. It was shown that in the absence of Polη, its closest paralogue, Polι, bypasses UV induced photoproducts and functions in a manner that delays the onset of skin cancer in mice^30,31^. Both Polη and Polι can copy a template with the most common oxidative DNA lesion, 8-oxoG. However, Polι does so by utilizing its ability for Hoogsteen pairing^32,33^. Additionally, both of the polymerases can, to some extent, participate in the bypass of lesions induced by methylating agents^34^. *In vitro* experiments have shown that Polι is capable of bypassing many other DNA lesions^33,35–38^, while *in vivo* a Polι deficiency has been suggested to sensitize cells to oxidative DNA damage^39^ and cause UV-induced mesenchymal tumorigenesis^40^.

As a consequence of a spacious active site, Y-family polymerases are more liberal than classical replicases in the selection of an incoming nucleotide, which together with a lack of proofreading activity, results in frequent mistakes when they synthesize DNA on an undamaged template^41^. Polι is the most mutagenic among all known human polymerases and its fidelity is strictly template-dependent^42,43^.

The recruitment of Y-family polymerases to the replication fork occurs as a result of complex regulation where protein posttranslational modifications and protein interactions play fundamental roles. All Y-family polymerases can interact through their dedicated domains with the replication processivity factor, PCNA. These interactions are additionally strengthened after DNA damage-induced PCNA monoubiquitination, as all Y-family polymerases are equipped with one or two, ubiquitin binding domains (UBMs or UBZs)^44,45^. Rev1 protein represents another interaction platform coordinating targeting of TLS polymerases to the blocked replication fork. In addition to protein-protein interactions, posttranslational modifications of Y-family polymerases seem to provide more specific instructions for their participation in TLS^2^. Consequently, it was shown that the modification status of Polη dramatically changes in response to DNA damage^46^. UV irradiation produces CPD dimers, the cognate substrate of Polη, and results in changes to the ubiquitination, phosphorylation and GlcNAcylation status of the TLS polymerase, which subsequently affects its recruitment to a stalled replication fork, CPD bypass and removal from DNA after TLS, respectively^46–51^. The modification of Polι caused by DNA damage has not been reported so far. In contrast to Polη, the monoubiquitination status of Polι does not change in response to DNA damaging agents, but the extent of polyubiquitination increases significantly after treatment with 1,4-naphthoquinones^25^. We have previously presented evidence that menadione-induced Polι polyubiquitination does not occur due to oxidative damage, but rather the inhibition of p300/CBP acetyltransferase activity^25^.

In the current work, we have shown that Polι is acetylated by the p300/CBP acetyltransferases and that this response is especially strong in cells treated with the S_N_2 alkylating agents, methyl methanesulphonate (MMS) and dimethyl sulfate (DMS), but not other DNA damaging treatments. We have identified the key protein interactions responsible for this modification and pinpointed the primary residue in Polι that is acetylated. Interestingly, while we found that the response to S_N_2 alkylating agents targets Polι for acetylation, the same is not true for its closest paralogue, Polη, suggesting a differentiation of cellular functions of these TLS polymerases. The possible consequences of acetylation on the cellular function(s) of Polι are discussed.

## Results

### p300 Interacts with Polι

**Figure 1:**
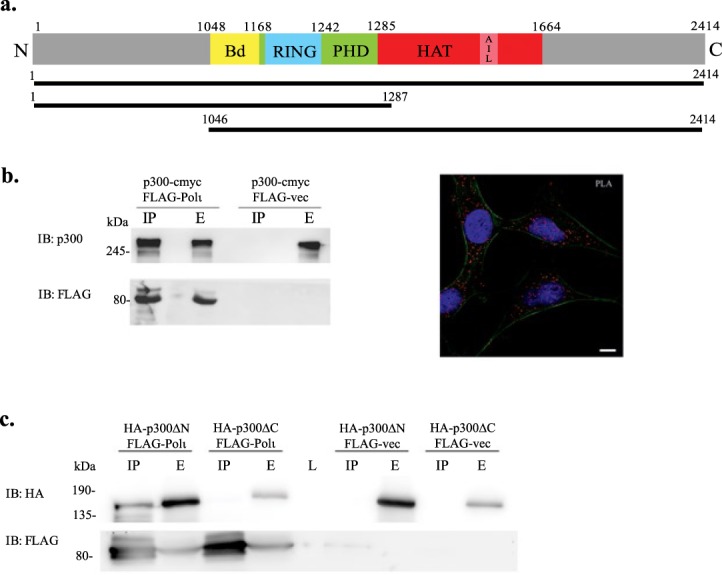
Mapping the region of p300 that interacts with Polι. (**a**) Schematic representation of p300 deletion constructs. The colorful block represents some of the important domains in p300: Bd - bromodomain, RING - non-canonical RING domain characteristic for ubiquitin ligases, PHD - plant homodomain, HAT - acetyltransferase catalytic domain, AIL -autoinhibitory loop. The amino acid location of the various domains are indicated above the block scheme. Constructs used are shown below. (**b**) Interaction between Polι and p300. Left panel shows the results of immunoprecipitation assays (IP) performed with anti-FLAG resins incubated with extracts (E) from cells co-expressing p300-c-myc and FLAG-Polι or p300-c-myc and empty FLAG vector as a negative control. Right panel shows the results of the proximity ligation assay (PLA), signal (red) provides evidence that endogenous Polι and p300 are close together in the cell, actin cytoskeleton is shown in green (Alexa488 Phalloidin staining), nucleus in blue (Hoechst staining). Scale bar is 10 µm. (**c**) Immunoprecipitation assays (IP) were performed with anti-FLAG resins that were incubated with extracts (E) from cells expressing FLAG-Polι or FLAG empty vector as a negative control and either of HA-tagged p300 deletion constructs p300ΔN or p300ΔC.

Our previous results suggested a link between Polι polyubiquitination and inhibition of the lysine acetyltransferase activity p300^25^, a large, multi-domain protein (Fig. 1a) influencing multiple processes in the cell [reviewed in^12^]. To further corroborate the relationship between Polι and p300, we initially investigated whether p300 physically interacts with Polι. We confirmed the interaction by performing FLAG co-immunoprecipitation assays in HEK293T cells transiently transfected with FLAG-Polι and a p300-c-myc expression vector (Fig. 1b). Additionally, we have also demonstrated the interaction between endogenous p300 and Polι proteins by performing a proximity ligation assay (PLA) (Fig. 1b).

Because p300 can both acetylate and ubiquitinate substrates^16^, we next examined whether the RING ubiquitin ligase domain, which is present in the p300 protein, or the HAT domain, which is responsible for the acetyltransferase activity of p300, is required for the interaction with Polι. The catalytic regions of p300 form a compact module in which the RING domain is juxtaposed with the HAT domain. We performed immunoprecipitation experiments with transiently transfected HEK293T cells using either of the two HA-tagged p300 deletion constructs and FLAG-tagged Polι. p300ΔC (amino acids 1–1287) lacking the HAT domain failed to interact with Polι, while p300ΔN (amino acids 1046–2414) interacted strongly with the protein, indicating that C-terminal half of the p300 protein, including the HAT domain, is required for binding to Polι (Fig. 1c).

**Figure 2:**
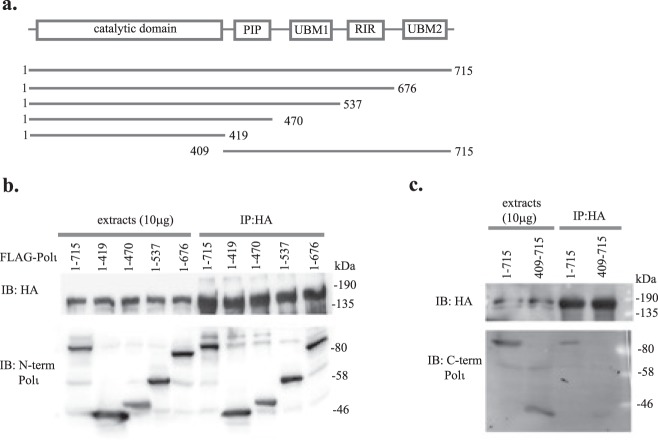
Mapping the region of Polι that interacts with p300. (**a**) Schematic representation of Polι with indicated domains, and Polι deletion constructs. (**b**) Immunoprecipitation assays were performed with 10μl of anti-HA resins that were incubated with equal amounts of extracts from cells co-expressing HA-p300ΔN and full-length FLAG-Polι or FLAG-Polι with deletions of various protein interacting domains. (**c**) Immunoprecipitation assays were performed with 10μl of anti-HA resins that were incubated with equal amounts of extracts from cells co-expressing HA-p300ΔN and full-length FLAG-Polι or FLAG-Polι lacking the N-terminal catalytic domain (IP- immunoprecipitation). In (**b**,**c**) the input controls contain 20μg or 10μg of protein extracts, respectively.

To further map the interaction between Polι and p300 and identify a region in Polι necessary for an interaction with the p300 protein, we generated a series of Polι deletion constructs lacking domains involved in protein-protein interactions including the ubiquitin binding motifs UBM1 and UBM2, the Rev1-interacting region (RIR) and the PCNA interacting peptide (PIP) (Fig. 2a). We used the HA-immunoprecipitation assay in HEK293T cells transiently transfected with HA-tagged p300ΔN and full-length, or truncated versions of Polι. As shown in Fig. 2b, HA-p300ΔN interacted with all of the Polι constructs lacking the known protein interacting domains, which suggests that the Polι catalytic domain located at the N-terminus of the protein is sufficient for the interaction with p300. To verify this hypothesis, we tested the interaction of HA-p300ΔN with Polι lacking the catalytic domain, but possessing all known protein-interacting regions (409–715aa). The recombinant Polι protein lacking its N-terminus did not interact with p300, implying that the presence of the catalytic domain of Polι is required for its interaction with the p300 protein (Fig. 2c).

### Polι is Acetylated *in vivo* and *in vitro*

**Figure 3:**
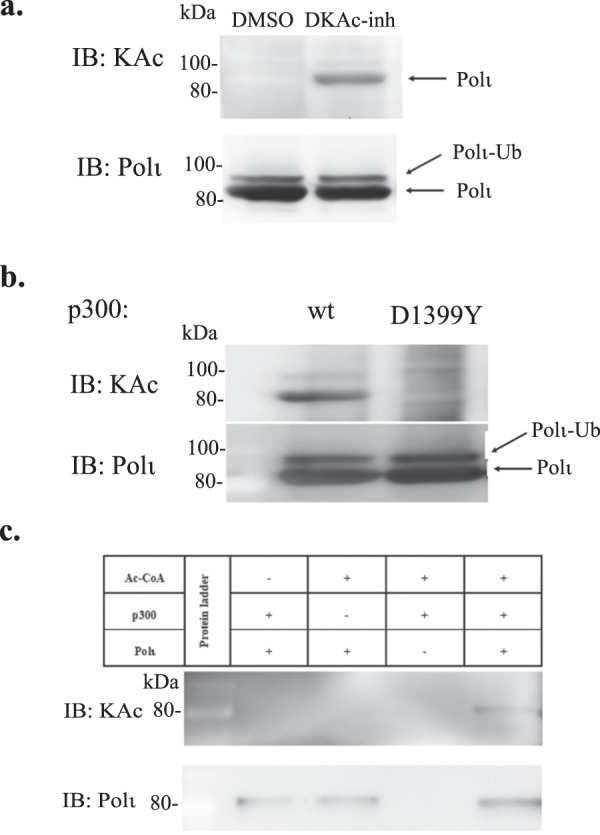
*In vivo* and *in vitro* acetylation of Polι by p300. (**a**) Immunoprecipitation assay of extracts from HEK293T cells co-transfected with FLAG-Polι and wild-type p300-c-myc. As indicated, prior to harvesting, cells were treated either with DMSO or with deacetylase inhibitor (DKAc) cocktail. (**b**) Immunoprecipitation assay used extracts from HEK293T cells co-transfected with FLAG-Polι and wild-type p300-c-myc, or FLAG-Polι and p300 containing the D1399Y mutation that abolishes the acetyltransferase activity of p300. 24hrs prior to harvesting, cells were treated with DKAc inhibitor. In panels (**a**,**b**), Polι acetylation was verified by western blot using antibodies against acetylated lysines (KAc). Polι was visualized using polyclonal rabbit antibodies against a C-terminal fragment of Polι. (**c**) *In vitro* acetylation of recombinant His-tagged Polι by p300. The reaction products were resolved by SDS-PAGE and analyzed by western blot using antibodies against KAc and against Polι.

Our current studies show that Polι directly binds the C-terminal half of p300 protein including the HAT domain. This interaction prompted us to test whether Polι is acetylated by p300 *in vivo* and *in vitro*. As demonstrated in Fig. 3a, acetylation of FLAG-tagged Polι was clearly detected in an immunoprecipitated (IP) complex using anti-FLAG resins, when cells co-expressing FLAG-Polι and p300-c-myc were pre-treated for 24 h with a deacetylation inhibitor (DKAc) cocktail, in contrast to cells treated with DMSO. To verify whether p300 is responsible for Polι acetylation, we co-transfected FLAG-Polι with either full-length wild-type p300 protein, or a variant with a D1399Y mutation that reduces acetyltransferase activity^52^. The acetylation of Polι decreased dramatically when co-expressed with the p300 D1399Y mutant with the acetyltransferase-deficiency, suggesting that Polι can be acetylated by p300 (Fig. 3b). In addition, we proved that Polι can be acetylated by p300 by performing *in vitro* experiments, in which full-length p300 was incubated with a purified His-tagged Polι protein and acetyl coenzyme A (Fig. 3c).

### Identification of Polι Acetylation Sites

**Figure 4:**
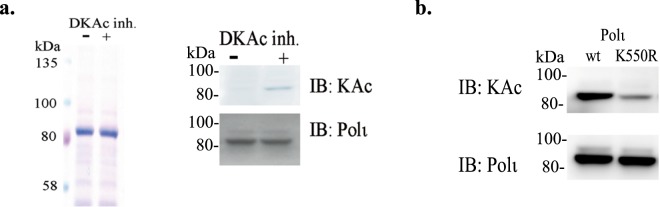
Identification of acetylation sites in Polι. (**a**) Coomassie Brilliant Blue-stained gel (left panel) of FLAG-tagged Polι purified from HEK293T cells transfected with the FLAG-Polι plasmid and treated, or untreated, with DKAc inhibitor cocktail as indicated. Polι acetylation of purified Polι was visualized by western blot (right panel) using monoclonal antibodies against acetylated lysines (KAc). Polι was visualized using polyclonal rabbit antibodies against a C-terminal fragment of Polι. (**b**) Effect of the K550R substitution on Polι acetylation. Western blot using monoclonal antibodies against acetylated lysines. Polι was visualized using polyclonal rabbit antibodies against a C-terminal fragment of Polι.

To further explore acetylation of Polι, we performed a series of mass spectrometry analyses to identify residues of Polι that are acetylated *in vivo* and in *in vitro*. We initially used anti-FLAG resins to purify FLAG-tagged Polι from HEK293T transiently transfected with the FLAG-Polι plasmid. These cells were then incubated with, or without, DKAc inhibitors for 24 hours (Fig. 4a) before the purified proteins were run on an SDS-PAGE gel and analyzed by mass spectrometry. Additionally, small amounts of the purified proteins were evaluated by western blot using antibodies against acetylated lysines and Polι. Compared to Polι isolated from untreated cells, we observed a faint acetylation signal from FLAG-Polι purified from cells treated with the DKAc inhibitor cocktail. In agreement with the western blot results, mass spectrometry analysis did not reveal any acetylated residues in untreated cells, whereas K550 was identified as the main acetylation site when cells were treated with DKAc inhibitors. In the Polι protein that was acetylated *in vitro*, we identified four acetylated lysines: K138, K440, K550 and K715 (Fig. S1). K550 is located in the RIR domain that is responsible for Polι’s interaction with Rev1, and was the only residue identified in both approaches. To determine if it is the primary site of acetylation, we co-transfected human HEK293T cells with a plasmid expressing p300 and a recombinant plasmid carrying wild-type FLAG-tagged Polι, or one containing a K550R substitution. Transfected cells were treated with DKAc inhibitor cocktail. Proteins pulled-down with anti-FLAG resin were then probed with antibodies against acetylated lysines. In the K550R mutant, the acetylation signal was significantly reduced (Fig. 4b), but still observable, thereby confirming that K550 is the primary acetylation site, but also suggesting the existence of alternate acetylation site(s) *in vivo*.

### Interaction of Acetylated Polι with Rev1

**Figure 5:**
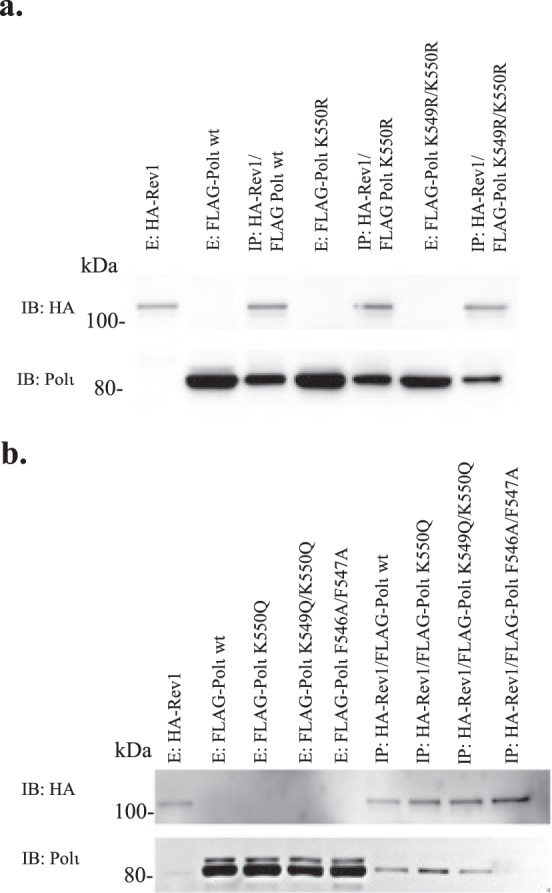
Effect of mutating Polι K550 residue and its interaction with Rev1. (**a**) Immunoprecipitation of Rev1 and wild-type Polι, or Polι K550 mutants. Immunoprecipitation assays were performed with HA-tagged full-length Rev1 immobilized on anti-HA resins that were incubated with extracts from cells expressing FLAG-tagged wild-type Polι, or Polι carrying single K550R, or double K549R/K550R mutations. (**b**) Immunoprecipitation of Rev1 and wild-type Polι, or Polι mutants mimicking K550 acetylation. Immunoprecipitation assays were performed with HA-tagged full-length Rev1 immobilized on anti-HA resins that were incubated with extracts from cells expressing FLAG-tagged wild-type Polι, or Polι carrying single K550Q, or double K549Q/K550Q mutations. As a control, the lack of interaction between Rev1 and Polι F546A/F547A is shown. E-extract, IP- immunoprecipitation.

Our observation that K550, which is located in the RIR domain, is the major acetylation site in Polι protein prompted us to check the role of the K550 residue itself and its acetylated form in the interaction of Polι with Rev1. Using anti-HA resins, we performed immunoprecipitation assays on extracts from HEK293T transiently transfected with plasmids encoding HA-tagged full-length Rev1 and either wild-type FLAG-Polι, or a variant carrying a single point mutation at K550. K549 is adjacent to K550 and it is known that if the primary residue is unavailable for posttranslational modification, the neighboring sites could be modified^46^. To determine if K549 might compensate for K550 if it is unavailable for acetylation, we also tested whether a double point mutation of K549 and K550 influences Polι’s interaction with Rev1. Initially, we tested lysine-to-arginine (K → R) mutants. Both, the single and double mutant of Polι maintained the interaction with Rev1 (Fig. 5a). To study the effect of the acetylation on the Polι-Rev1 interaction, we replaced the lysine residues with glutamine, since previous studies have suggested that glutamine substitutions simulate acetylation^53,54^. Immunoprecipitation experiments with K → Q Polι mutants indicate that there is no effect on Polι’s ability to interact with Rev1 (Fig. 5b). As a control for these experiments, we used an F546A/F547A Polι mutant, that has been shown to abolish the interaction between Polι and Rev1^55^. Together, our results suggest that K → R mutations, which simulate an unmodified state, or K → Q mutations, which mimic acetylation, do not significantly change the interaction between Polι with Rev1.

### Polι Acetylation in Response to a Variety of DNA Damaging Agents

**Figure 6:**
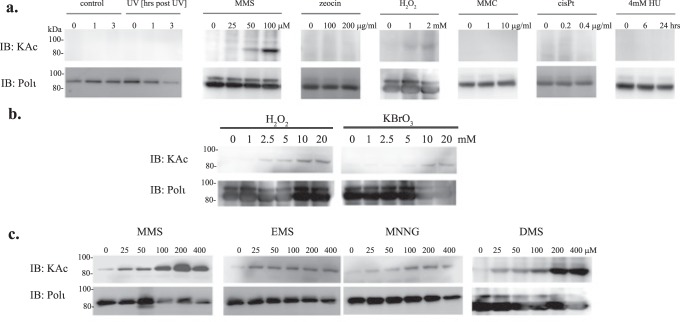
Effect of DNA-damaging agents on Polι acetylation in HEK293T cells. (**a**) Acetylation of Polι in HEK293T cells transfected with the FLAG-Polι expressing vector and treated, as indicated, with UV-irradiation – 7 J/m^2^, MMS – 1 h, zeocin – 12 hrs, hydrogen peroxide (H_2_O_2_) – 1 h, mitomycin C (MMC) – 40 hrs, cisplatin (cisPt) – 12 h, or hydroxyurea (HU). 10μg of protein extract was loaded in each line. Polι acetylation was visualized by western blot using monoclonal antibodies against acetylated lysines (KAc). Polι was visualized using polyclonal rabbit antibodies against a C-terminal fragment of Polι. (**b**) Acetylation of Polι in HEK293T cells transfected with FLAG-Polι plasmid and treated for 1 h with increasing doses of the oxidative agents, Hydrogen peroxide (H_2_O_2_) or Potassium bromate (KBrO_3_). (**c**) Acetylation of Polι in HEK293T cells transfected with the FLAG-Polι plasmid and treated for 1 h with alkylating agents, MMS, EMS, MNNG and DMS.

The Polι acetylation observed in the previous experiments was mostly detected in cells overexpressing the p300 protein. To determine the physiological conditions under which Polι is acetylated we tested acetylation of the ectopically expressed FLAG-Polι in cells with native levels of p300, after treatment with a variety of DNA damaging agents. We investigated the influence of UV irradiation, which induces cyclobutane pyrimidine dimers and other photoproducts; MMS, a source of alkylating damage; zeocin stimulating DNA double strand breaks; hydrogen peroxide, causing DNA oxidative lesions and two DNA crosslinking agents, used in chemotherapy, mitomycin C and cisplatin. Additionally, we have also tested whether hydroxyurea, which blocks replication fork progression due to decreased dNTPs production, has any influence on the acetylation status of Polι. As shown in Fig. 6a, exposure of cells to most of the DNA damaging agents and hydroxyurea did not result in significant Polι acetylation. In contrast, MMS treatment substantially induced Polι acetylation. We also observed a weak Polι acetylation signal in hydrogen peroxide treated cells. To further analyze the phenomenon of Polι acetylation in response to MMS and hydrogen peroxide, we tested whether other alkylating and oxidizing agents were also able to induce Polι acetylation. We exposed HEK293T cells to increasing concentrations of hydrogen peroxide and potassium bromate, which is known to cause DNA oxidative lesions (Fig. 6b). Both oxidative agents, at higher doses, caused some acetylation of Polι. In comparison to MMS, we investigated the influence of other alkylating agents, EMS and MNNG, on Polι acetylation (Fig. 6c). Both alkylating agents caused some acetylation of Polι that was much weaker than MMS. Since alkylation by MMS proceeds *via* second-order nucleophilic substitution (S_N_2) and MNNG is an S_N_1 alkylating agent, we examined acetylation of Polι with DMS, another S_N_2 alkylating agent. Interestingly both MMS and DMS induced a much higher acetylation level than EMS and MNNG. It is known that compared to MMS, much lower concentrations of DMS induce the same amount of alkylation damage^56^, which might explain the difference in the strength of the acetylation signal when using equimolar amount of MMS and DMS.

### Characterization of MMS-induced Polι acetylation

**Figure 7:**
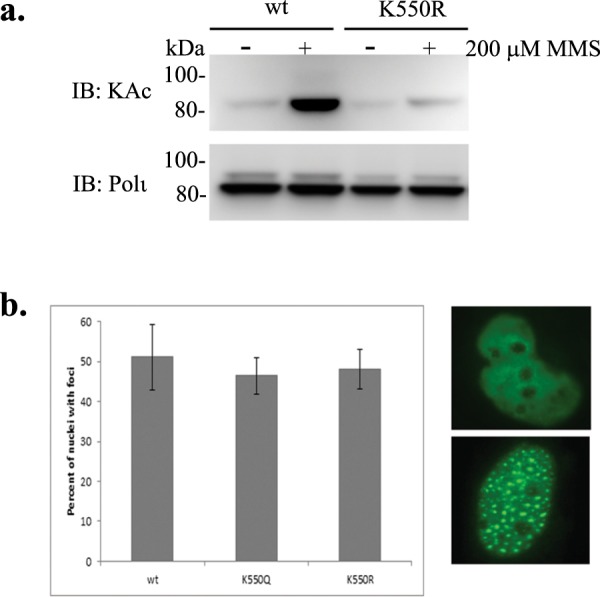
Characterization of Polι K550R and K550Q variants. (**a**) Effect of the K550R substitution on MMS-induced Polι acetylation. Western blot using monoclonal antibodies against acetylated lysines. Polι was visualized using polyclonal rabbit antibodies against a C-terminal fragment of Polι. (**b**) Comparison of Polι localization into “replication factories” of wild-type GFP-Polι, GFP-Polι-K550R, and GFP-Polι-K550Q, respectively that block, or mimic, acetylation at the K550 residue. Respective plasmids were transfected into MRC5 cells. Histogram showing the percentage of nuclei with foci (left panel). Numbers are based upon 3 replicates where 200 nuclei were counted for each cell line from GFP positive cells. Example of GFP-positive cells with a nucleus without foci (right top panel), or with foci (right bottom panel).

To further corroborate MMS-induced Polι acetylation, we used mass spectrometry analysis to identify Polι acetylation sites in cells exposed to MMS. HEK293T cells transiently transfected with the FLAG-Polι plasmid were exposed to 200 μM MMS for 1 h. In a control sample from cells not treated with MMS, K550 was again the only acetylated residue. The analysis of Polι protein isolated from MMS-exposed cells also revealed acetylation of K550 and an additional three other lysine residues; K440, K446 and K530 (Fig. S2). Next, by using the K550R mutant, we examined whether K550 is an important site for MMS-induced Polι acetylation. Indeed, MMS-stimulated Polι acetylation was drastically reduced when K550 was unavailable for modification (Fig. 7a). We conclude that K550 is, in general, the main Polι acetylation site after MMS treatment. To test the *in vivo* properties of the acetylated and unacetylated protein, we examined if Polι variants with K550R and K550Q substitutions could accumulate in DNA replication foci similar to the wild-type protein. Our results show that mutations that either block posttranslational modification(s) at K550 (K550R) or mimic its acetylation (K550Q), do not alter the accumulation of Polι into “replication factories” (Fig. 7b) suggesting that acetylation functions in fine tuning, rather than in the general regulation of Polι’s cellular activities. We also speculate that, since the main acetylation site is located outside the catalytic domain of Polι, it is unlikely to directly affect Polι’s enzymatic activities. We do not however, exclude that it might indirectly influences enzymatic activities of the polymerase via interactions with other proteins, and this will be a subject of future investigation.

**Figure 8:**
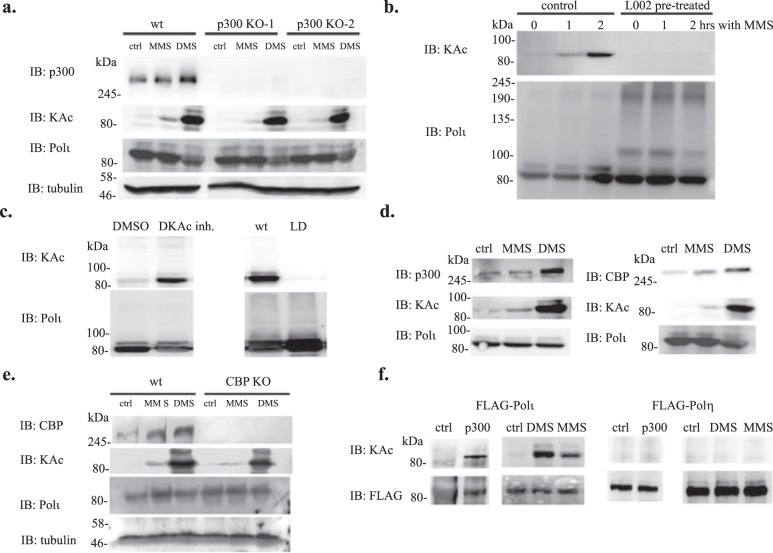
Characterization of Polι acetylation. (**a**) Effect of a p300 knock-out on Polι acetylation in cells treated for 1 h with 200 μM MMS or DMS. (**b**) Effect of a p300/CBP inhibitor, L002, on MMS-induced Polι acetylation. (**c**) Immunoprecipitation assay on extracts from HEK293T cells co-transfected with FLAG-Polι and wild-type mCBP, or with an L1435A/D1436A (LD) mutation that abolishes acetyltransferase activity of CBP. (**d**) Effect of 1 h treatment with 200 μM MMS, or DMS, on the cellular level of p300 and CBP. (**e**) Effect of CBP knock-out on Polι acetylation in cells treated for 1 h with 200 μM MMS or DMS. (**f**) Effect of p300 overproduction, or MMS, or DMS treatment on Polι or Polη acetylation. Immunoprecipitation of FLAG-tagged polymerases from extracts from HEK293T cells transfected with FLAG-Polι (left panel) or FLAG- Polη (right panel). As indicated, cells were co-transfected with wild-type p300-c-myc, and treated for 1 h with 200 mM DMS, or MMS. Unless otherwise stated, HEK293T cells were transiently transfected with the FLAG-Polι plasmid. 24 hrs post transfection cells were treated for 24 hrs with DKAc inhibitor cocktail. Proteins were visualized by western blot using the following antibodies; polyclonal antibodies against p300 (p300 protein); monoclonal antibodies against acetylated lysines (acetylated Polι); polyclonal rabbit antibodies against a C-terminal fragment of Polι; or monoclonal antibodies against the FLAG epitope, as indicated.

The acetylation of Polι in response to MMS treatment was observed without ectopic expression of p300. This raised the question of whether p300 itself, or other acetyltransferases modify Polι under alkylating conditions. To establish whether p300 acetyltransferase is responsible for the modification, we disrupted the p300 gene in a HEK293T cell line using CRISPR-Cas9 technology and assayed the acetylation of Polι in response to MMS and DMS in the p300 knock-out cells. To our surprise, in two independently isolated p300 knock-out cell lines that were transiently transfected with the FLAG-Polι plasmid, MMS/DMS-induced acetylation of Polι was still observable (Fig. 8a), suggesting that p300 is not the only acetyltransferase responsible for Polι’s modification. In parallel, we examined Polι acetylation following MMS treatment in cells exposed to the p300 acetyltransferase inhibitor, L002. HEK293T cells transfected transiently with FLAG-Polι were pre-incubated for 24 hrs with DKAc inhibitor and subsequently exposed, or not (controls), to L002. After a 1 hour exposure, cells were treated with MMS for an additional one, or two hours. Polι acetylation was assessed in extracts collected at the various time points. As shown in Fig. 8b, Polι acetylation is only noticeable in extracts from cells that were not treated with p300 inhibitor, strongly suggesting that incubation of cells with L002 effectively prevented Polι acetylation. These findings imply that p300 is accountable for Polι acetylation in response to MMS exposure. However, in human cells, p300 has a paralogue, CBP, and together they are the only members of one of several classes of acetyltransferase families. It is known that to some extent L002 inhibits CBP acetyltransferase activity and p300 and CBP often acetylate the same substrates, albeit with different potency^16,57^. To verify that CBP can acetylate Polι, we assayed Polι acetylation in HEK293T cells co-transfected with the plasmid carrying FLAG-tagged Polι and a plasmid carrying murine CBP. In the cells treated for 24 hrs with DKAc inhibitor, Polι acetylation was clearly observable (Fig. 8c). To further confirm the ability of CBP to acetylate Polι, we assayed cells co-transfected with FLAG-tagged Polι and either wild-type CBP, or the inactive *L1435A*/*D1436A* mutant^58^. In cells expressing the HAT catalytic dead CBP mutant, Polι was not acetylated, thus confirming that CBP also acetylates Polι (Fig. 8c). Finally, we disrupted the CBP gene in a HEK293T cell line using CRISPR-Cas9 technology and verified acetylation of Polι in response to MMS and DMS in the CBP knock-out cells (Fig. 8d). Similar to the p300 KO cells, acetylation of Polι was still observable. Together, our results suggest that both p300 and CBP acetyltransferases can acetylate Polι.

As both over-expression of p300/CBP from a plasmid, or MMS/DMS treatment resulted in increased Polι acetylation, we decided to check whether treatment with S_N_2 alkylating agents influences the levels of the p300/CBP acetyltransferases. In our experiment, we examined the level of native p300 and CBP protein in cells treated with MMS, or DMS. It appears that the amount of p300 protein is slightly increased in MMS treated cells and a stronger effect is observable in cells treated with DMS. Detection of an augmented amount of acetyltransferase in response to those S_N_2 alkylating agents is clearer in the case of CBP (Fig. 8e).

Finally, our observation that Polι undergoes p300/CBP-dependent acetylation in response to S_N_2 alkylating agents prompted us to determine whether this phenomenon is shared with other Y-family polymerases, particularly with Polη, which is the closest paralogue of Polι in mammalian cells. We investigated acetylation of Polη in cells transfected with FLAG-tagged Polη over-expressing p300 or treated with alkylating agents. It appears that under the conditions where we observe significant acetylation of Polι there is no acetylation of Polη, suggesting that this regulatory mechanism is specific for Polι (Fig. 8f).

## Discussion

Polι is a TLS polymerase present in mammals, some other vertebrates and some fungi. The cellular function of the enzyme is still far from being understood. Despite the fact that it was identified two decades ago^59^ and possesses very peculiar features that are not found in other Y-family DNA polymerases, such as a dRP-lyase activity and an extremely high mutagenic potential when copying template T, the cellular function of Polι has yet to be determined^42,60^. In an attempt to decipher the circumstances requiring such an enigmatic enzyme, we investigated the mechanism of Polι’s regulation. Polι, like other Y-family polymerases, seems to be primarily controlled by the combined action of posttranslational modifications and protein-protein interactions. Similar to other Y-family DNA polymerases, Polι is monoubiquitinated *in vivo*, and so far, it is only known that such a modification facilitates its physical and functional interaction with Polη^61^. Mass spectrometry analysis previously identified over 27 unique ubiquitination sites in Polι, localized in different functional domains^25^. Additionally, our earlier studies showed that inhibition of p300 acetyltransferase activity induces Polι polyubiquitination^31^. In the current manuscript, we show that Polι not only directly interacts with the p300 acetyltransferase but is subject to acetylation itself (Fig. 3). p300 has two enzymatic activities, an acetyltransferase activity, associated with its HAT domain, and a ubiquitin ligase activity connected with a non-canonical RING domain^15^. We explored whether either of these domains is located in the region required for an interaction with Polι. Our results revealed that Polι does not interact with the N-terminal half of p300 containing the RING domain, but rather with the C-terminal part of p300, including the HAT domain (Fig. 1). Although, we cannot formally exclude the possibility that Polι can also be ubiquitinated by p300, the lack of an interaction with the N-terminal part of the protein that is required for p300 E3 autoubiquitination and E4 polyubiquitination of p53 suggests that p300 ubiquitination of Polι is unlikely.

The main acetylation site of Polι is K550 located in Polι’s Rev1 interacting region (RIR motif 539-ASRGVLSFFSKKQMQD-554). K550 is a conserved residue found in many different species of mammals, but not other vertebrates, which correlates with the conservation of the RIR (Fig. S3). Moreover, K550 is conserved in all human and mouse paralogs of Polι, Polη and Polκ^62^. Nonetheless, we demonstrated that both K550R or K550Q mutations, respectively blocking and mimicking acetylation, do not significantly affect the ability of Polι to interact with Rev1. This result is consistent with previous results defining the RIR motif as x-x-x-F-F-y-y-y-y, consisting of two consecutive phenylalanine residues proceeded by no specific residues (x) and followed by any four residues (y) excluding proline^55^. On the other hand, Liu *et al*., observed significant reduction in the strength of the interaction between Rev1 and Polκ when the corresponding lysine in Polκ was mutated to glycine (K571G)^63^. Lysine 550 is located near the interface with Rev1 and can also serve as one of many possible ubiquitination sites^25^. In our previous studies, we speculated that attaching ubiquitin to K550 would obstruct the interaction of Polι and Rev1^25^. Following this idea, we can hypothesize that acetylating K550 might prevent its susceptibility to ubiquitination, thereby assuring an opportunity for the two polymerases to interact. In general, the RIRs of TLS DNA polymerases play an essential role in regulating TLS activity, however, the interaction between the C-terminal domain of Rev1 and RIR motifs of Polη, Polι and Polκ are not exceptionally tight and are often competitive with one another. So, it seems that a Rev1-RIR interaction results from a dynamic association, rather than a stable connection. Even though the results of our immunoprecipitation experiments show that in general, blocking, or mimicking, Polι acetylation does not notably alter the interaction with Rev1, we cannot exclude the possibility that it can influence polymerase engagement in the microenvironment of a particular replication block.

To further corroborate the physiological conditions leading to Polι’s modification, we investigated the acetylation in response to a variety of DNA damaging agents. The results of our experiments showed that Polι is acetylated in response to alkylating agents and in much lower extent to oxidative agents (Fig. 6).

It is worth noting that similar to Polι acetylation, Lee *et al*., observed global acetylation of proteins in cells exposed to genotoxic agents, particularly MMS and other alkylating agents and to a lower extent to hydrogen peroxide^64^. However, in contrast to the global protein acetylation that, according to Lee *et al*., does not depend on p300/CBP, our results show that these two acetyltransferases are responsible for Polι acetylation (Figs 3 and 8). Additionally, our results suggest that, at least partially, increased Polι acetylation in response to MMS and DMS could be a consequence of higher cellular levels of the two acetyltransferases, as we observed slight induction of both p300 and CBP in MMS or DMS treated cells (Fig. 8d). We are unaware of any information in the literature reporting the inducibility of p300/CBP in response to DNA damaging agents. However, Cohen *et al*., showed that CBP protein accumulates in the cytoplasm in response to UV radiation^65^.

Although, our results point to MMS/DMS as the main facilitator of Polι acetylation, we noticed that oxidative agents (hydrogen peroxide and potassium bromate) can also induce Polι acetylation (Fig. 6b), but to a much lower extent. Since both alkylation and oxidation of DNA, as well as other DNA damaging treatments used in this research, can activate the ATR/ATM dependent cascade of kinases governing DNA damage response (DDR), one can speculate that Polι acetylation reflects the level of DDR induction. However, based on the literature data on phosphorylation of the landmarks of DDR: Chk1^66^, Chk2^67^ or γH2AX^68^, in response to the DNA damaging treatments inducing or not Polι acetylation, we conclude that the Polι acetylation does not correlate with activation of DDR. While the exact mechanism of how Polι is influenced by the cellular responses is not known, it was previously shown that Polι can bypass alkylation or oxidative lesions both *in vitro* and *in vivo*^32–34,39,69–73^.

Both alkylation and oxidative DNA lesions are substrates of base excision repair (BER). It is also worth mentioning that Polι displays BER activity *in vitro* and *in vivo* and functionally interacts with the BER scaffold protein XRCC1^39,60,74^. Furthermore, Polι possesses deoxyribose phosphate (dRP) lyase activity, a feature characteristic for BER polymerases^58,68^. Strikingly, proteins involved in BER represent a significant group among all proteins involved in DNA damage response that were found to be acetylated^5,75–78^. This suggests that protein acetylation can considerably influence the activity of BER enzymes, even though it cannot be unequivocally proven that it activates or represses BER.

Interestingly, the level of Polι acetylation is particularly high in cells treated with two S_N_2 alkylating agents: MMS and DMS, in contrast to rather moderate acetylation in response to EMS (S_N_1/S_N_2) and MNNG (S_N_1). One could speculate that the DNA lesions produced by S_N_2 rather than S_N_1 alkylating agents might serve as a signal for Polι acetylation. The stronger response to S_N_2 than S_N_1 alkylators is intriguing, since the sites methylated in an S_N_2 reaction in duplex DNA are also modified by S_N_1 alkylating agents. However, in single-stranded DNA, some sites are more reactive with MMS than with MNNG^79^. Accordingly, DNA repair dioxygenases from the AlkB family cause resistance to cell killing by S_N_2 but not the S_N_1 alkylating agents^80–82^. On the other hand, S_N_2 and S_N_1 alkylating agents differently modify amino acids residues^83^; therefore, at the moment we cannot exclude the possibility that Polι acetylation results from S_N_2 alkylation of proteins, or other cellular constituents. However, in light of the known DNA damage tolerance/repair activities of TLS polymerases, the functional connection of Polι acetylation with DNA rather than protein modifications by S_N_2 alkylating agents seems more credible. Nonetheless, this phenomenon needs to be investigated in detail and will be the subject of future studies.

Despite the fact that Polι, similar to many other cellular proteins, is acetylated in response to MMS, its closest paralogue, Polη, does not follow the same pathway. Additionally, it has been recently shown that Polκ, another member of the Y-family DNA polymerases, is not a substrate for *in vitro* CBP/p300 acetylation^22^. Polι was thought to be a less efficient and more error prone back-up polymerase for Polη^31,84^. Our results, however, suggest that despite many similarities between these two Y-family enzymes, there are cellular requirements and conditions devoted specifically for Polι and acetylation of the enzyme might play a role in adjusting its performance to these circumstances.

### Experimental procedures

*Reagents-* Were purchased from the following vendors: L002, plumbagin, MMS, EMS, MNNG, DMS, hydroxyurea, potassium bromate, acetyl coenzyme A, Hoechst from Sigma Aldrich; deacetylation inhibitor cocktail from Santa Cruz; zeocin from InvivoGen, MMC and cisplatin from TCI, hydrogen peroxide (Chempur), Anti DYKDDDDK Affinity Gel (Biotool), anti HA beads (Life Technologies), DMSO (BioShop), Turbofectin 8.0 from Origene, Alexa Fluor 488 Phalloidin (Thermo Fisher Scientific);

*Antibodies*- anti-FLAG (Abnova, MAB2094), anti-p300 (Santa Cruz, sc-585X and Abcam, ab54984), anti-HA (Abcam, ab9110 and Abnova, MAB7911), anti-Polι (Novus Biologicals, NB100-175), anti-N-terminal Polι and anti-C-terminal Polι^7^, anti-acetylated lysine (Thermo Scientific, MA1-2021), anti-CBP (Bethyl Laboratories, PLA0097 and Santa Cruz, sc-730), anti-tubulin (Abcam, ab 6160).

#### Plasmids

Vectors expressing truncated or mutated variants of Polι (pJRM258 – pJRM262) were generated by sub-cloning respective PCR fragments into pJRM46^61^ expressing N-terminal FLAG-tagged full-length human Polι. Plasmids pJRM264, pDH77, pGS11, pGS7 and pNWA10 are pJRM46 derivatives with single, or double lysine to arginine, lysine to glutamine or lysine to alanine substitutions that were generated by chemically synthesizing appropriate DNA fragments (Genscript) and subsequently sub-cloned into pJRM46. Vectors pJRM242 and pJRM244, expressing N- or C-terminal fragments of p300 proteins tagged with an HA-epitope, were generated by sub-cloning respective PCR fragments into the pCMV6AN-HA vector (Origene). Plasmid pGS23, expressing full-length wild-type human Rev1 was generated by sub-cloning the codon optimized synthesized *REV1L* gene (Genscript) into pCMV6AN-HA. Plasmids pGS24 and pGS25 expressing eGFP-tagged full-length Polι with single K550R, or K550Q mutations, respectively, were generated by chemically synthesizing appropriate DNA fragments (Genscript) and their subsequent sub-cloning into pDH24^85^. The full list of all the plasmids used in this study is shown in Table 1.

**Table 1:** Plasmids used in this study.

Plasmid	Description		Source/Ref.
pJRM46	pCMV6AN-DDK-polι	1-715aa	^61^
pJRM258	pCMV6AN-DDK-polι	1-419aa	this work
pJRM259	pCMV6AN-DDK-polι	1-470aa	this work
pJRM260	pCMV6AN-DDK-polι	1-537aa	this work
pJRM261	pCMV6AN-DDK-polι	1-676aa	this work
pJRM262	pCMV6AN-DDK-polι	409-715aa	this work
pJRM264	pCMV6AN-DDK-polι	K550R	this work
pDH77	pCMV6AN-DDK-polι	K550Q	Woodgate lab
pGS7	pCMV6AN-DDK-polι	K549R/K550R	this work
pGS8	pCMV6AN-DDK-polι	K549Q/K550Q	this work
pNWA10	pCMV6AN-DDK-polι	F546A/F547A	Woodgate lab
pJRM160	pCMV6AN-DDK-polη		this work
pDH24	peGFP-Polι		^89^
pGS24	peGFP-Polι K550R		this work
pGS25	peGFP-Polι K550Q		this work
pCMVβ-p300-myc			gift from Tso-Pang Yao (Addgene # 30489)
pCMVβ-p300.DY-myc			gift from Tso-Pang Yao (Addgene # 30489)
pJRM242	pCMV6AN-DDK-p300ΔC		this work
pJRM244	pCMV6AN-DDK-p300ΔN		this work
pcDNA3β-FLAG-CBP-HA			^58^
pcDNA3β-FLAG-CBP-LD-HA			^58^
pGS23	pCMV6AN-HA-Hs_CO-FL-Rev1		this work
pJM868	His-Ec-CO-polι_wt		^87^

#### Plasmid Transfection and Protein Expression

In all of our experiments, we have used ectopically expressed Polι, because the endogenous level of Polι in HEK293T cells is very low^86^.

Mammalian expressing constructs were transfected into HEK293T cells using Turbofectin 8.0 according to manufacturer’s instructions (Origene). Twenty-four to forty-eight hours post transfection cells were harvested and lysed. The presence of expressed proteins of interest was verified by western blot.

#### Human p300 and CBP Knock-out by CRISPR/Cas9

A p300 knock-out in a HEK293T cell line was generated using the p300CRISPR/Cas9 KO and p300HDR plasmids purchased from Santa Cruz (sc-400055 and sc-400055-HDR, respectively). A CBP knock-out in the HEK293T cell line was performed using CBP CRISPR/Cas9 KO and CBP HDR plasmids purchased from Santa Cruz (sc-400200 and sc-400200-HDR, respectively). The knock-out procedure was performed according to the manufacturer’s recommendations. Briefly, HEK293T cells were co-transfected with p300CRISPR/Cas9 KO and p300HDR or CBP CRISPR/Cas9 KO and CBP HDR plasmids and the successful co-transfections were visually confirmed by detection of the green fluorescent protein (GFP) and red fluorescent protein (RFP) via fluorescent microscopy. 48 hrs post transfection the media was removed and replaced with one containing puromycin and cells were selected on the medium with the antibiotic for another 5 days with fresh media replacement every 2 days. Next, single cell colonies were isolated and complete knockouts were confirmed by western blot with antibodies against p300 or CBP.

#### Foci Formation of DNA Polι

The GFP- Polι foci formation assay was performed as described previously^86^. Briefly, the eGFP constructs were transfected into MRC5 fibroblasts previously plated onto coverslips. Twenty-four hours after transfection, cells were fixed using 3.7% formaldehyde in PBS and mounted onto slides using Mowiol mounting medium. Fluorescence images of cell nuclei were acquired on a Zeiss Axiovert 40 CFL (Carl Zeiss) with X-cite Series 120Q light source, using Zen 2 (blue edition) software. A minimum of 200 GFP-expressing nuclei, were analyzed for each cell line per line.

#### Immunoprecipitation Assay

For the immunoprecipitation assay of acetylated Polι and Polη, respective cell extracts including FLAG-tagged proteins were incubated for one hour to overnight at 4 °C with Anti-DYDDDDK (Flag) Affinity Gel (Biotool), washed three times and analyzed directly by SDS-PAGE and western blot. For the immunoprecipitation assay regarding Polι interaction, respective cell extracts including FLAG- or HA-proteins were incubated for one hour at 4 °C with Anti-DYDDDDK (Flag) Affinity Gel (Biotool), or Pierce HA Epitope Tag Antibody Agarose (Thermo Scientific) respectively. Post incubation resins were washed five times and analyzed directly by SDS-PAGE and western blot.

#### Proximity Ligation Assay (PLA)

Prior to the proximity ligation assay (PLA), MRC5 fibroblasts were cultured and fixed on slides with 4% paraformaldehyde and 4% sucrose in PBS for 10 min at room temperature. After fixation, the cells were rinsed with PBS then incubated overnight at 4 °C with primary antibodies for Polι and p300 diluted 1:250 in 1% donkey serum (DS) and 0.2% Triton X-100 in PBS. The proximity ligation assay (PLA; Sigma DUO92004-100RXN) was performed using 40 μL of reaction per slide according to the manufacturer’s instruction. In brief, slides after overnight incubation with primary antibodies recognizing Polι and p300 were washed with PBS (3x) and incubated with two PLA secondary antibodies with probes, anti-mouse MINUS and anti-rabbit PLUS for 1 hour at 37 °C. Slides were then washed with buffer A, followed by the ligation reaction, for 30 min at 37 °C. Subsequently the amplification reaction was performed for 100 min at 37 °C, followed by washing with buffer B (all ingredients and buffers were provided by manufacturer) and mounting with VECTASHIELD Mounting Medium with DAPI for nucleus visualization. Images of immunofluorescently stained cells were acquired using confocal microscopy. The confocal system consisted of a Zeiss LSM800 Exciter microscope equipped with lasers that produced light at 405, 488, and 594 nm wavelengths was used for the acquisition of pictures of PLA positive cells, 40×/1.30 objective was used to scan samples. A series of continuous optical sections at 0.4 µm intervals along the z-axis of a cell were scanned for all fluorescent signals and stored as a series of 1024 × 1024 pixel images. The laser power and image acquisition settings were kept constant.

#### Protein Purification

Full-length recombinant human Polι was purified as described previously^87^. Briefly, pJM868 plasmid encoding His-tagged *Escherichia coli*-codon optimized human Polι^87^ was expressed in the *E*. *coli* strain RW644^88^. The His-Polι protein was purified on HisPur Ni-NTA Superflow Agarose (Thermo Scientific) as recommended by the manufacturer. The eluate containing Polι was dialyzed in buffer including 20 mM sodium phosphate pH 7.3, 10 mM sodium chloride, 10% glycerol, 10 mM 2-mercaptoethanol, and applied to HP Q Sepharose (GE Healthcare). Polι was eluted in a step gradient of sodium chloride. Full-length recombinant p300 was purchased from Active Motif.

#### Mass Spectrometry

LC-MS analysis of gel slices was conducted in Mass Spectrometry Lab, Institute of Biochemistry and Biophysics, Polish Academy of Science, where they were analyzed by mass spectrometry as a custom contract service.

#### *In vitro* Acetylation

Acetylation reactions were performed in acetylation buffer (50 mM Tris pH8, 150 mM sodium chloride, 1 mM DTT, 10 mM sodium butyrate, 5% glycerol) with the addition of 100 μM acetyl coenzyme A, 1 mM PMSF, 100 ng of p300 (Active Motif) and purified recombinant Polι for 1 hour at 30 °C. The reaction was then stopped by the addition of Laemmli buffer and followed by SDS-PAGE.

## Supplementary information

Supplementary Figures
