# Supplementary material for: DNA polymerase ι is acetylated in response to SN2 alkylating agents
Source: Sci Rep. 2019 Mar 18;9:4789. doi: 10.1038/s41598-019-41249-3 (PMC6423139; doi:10.1038/s41598-019-41249-3)
Supplement: Supplementary file 1 — Supplementary Figures [file 41598_2019_41249_MOESM1_ESM.pdf]

DNA polymerase ι is acetylated in response to S_N2 alkylating agents

Justyna McIntyre[1*], Aleksandra Sobolewska[1], Mikolaj Fedorowicz[1], Mary P. McLenigan[2], Matylda Macias[3], Roger Woodgate[2], Ewa Sledziewska-Gojska[1]

From the [1]Institute of Biochemistry and Biophysics, Polish Academy of Sciences, ul. Pawinskiego 5a, 2-106 Warsaw, Poland; [2]Laboratory of Genomic Integrity, National Institute of Child Health and Human Development, National Institutes of Health, Bethesda, MD 20892-3371, USA, [3]Laboratory of Molecular and Cellular Neurobiology, International Institute of Molecular and Cell Biology, ul. Ks. Trojdena 4, 02-109, Warsaw, Poland.

Running title: Polymerase iota acetylation

* To whom the correspondence should be addressed: Justyna McIntyre: Institute of Biochemistry and Biophysics, Polish Academy of Sciences, ul. Pawinskiego 5a, 2-106 Warsaw, Poland; jmcintyre@ibb.waw.pl; Tel.(+48) 22-592-11-15;

Keywords: Y-family DNA polymerase, polymerase iota, post-translational modification (PTM), ubiquitylation (ubiquitination), acetylation, p300/CBP, DNA damage, DNA polymerase

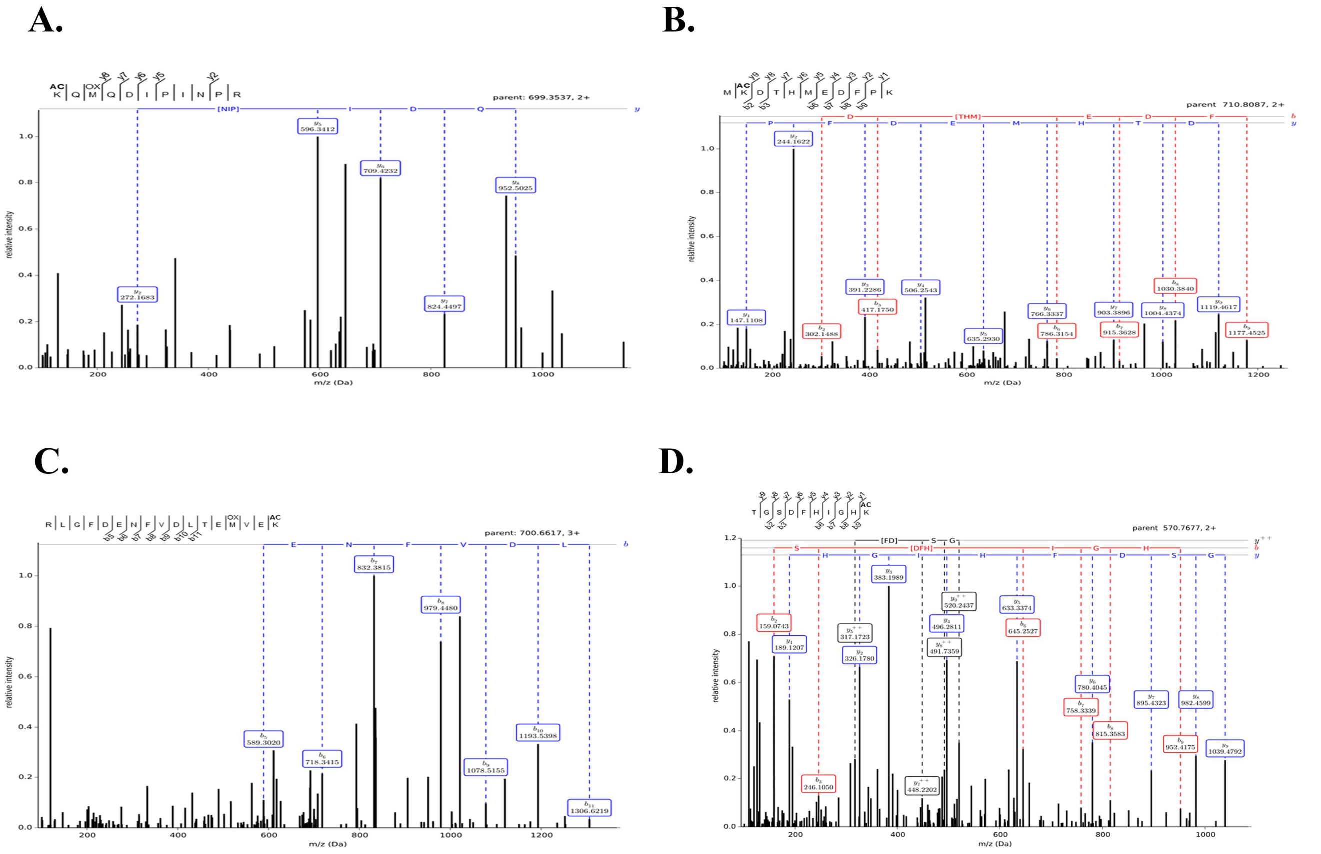

Figure S1. Mass spectrometry analysis of acetylated Polι protein recovered from HEK293T cells ectopically expressing p300. Fragmentation spectrum corresponding to respective peptides. Highlighted peaks correspond to b-series ions (blue) and y-series ions (red). Each spectrum of modified peptides was manually curated.

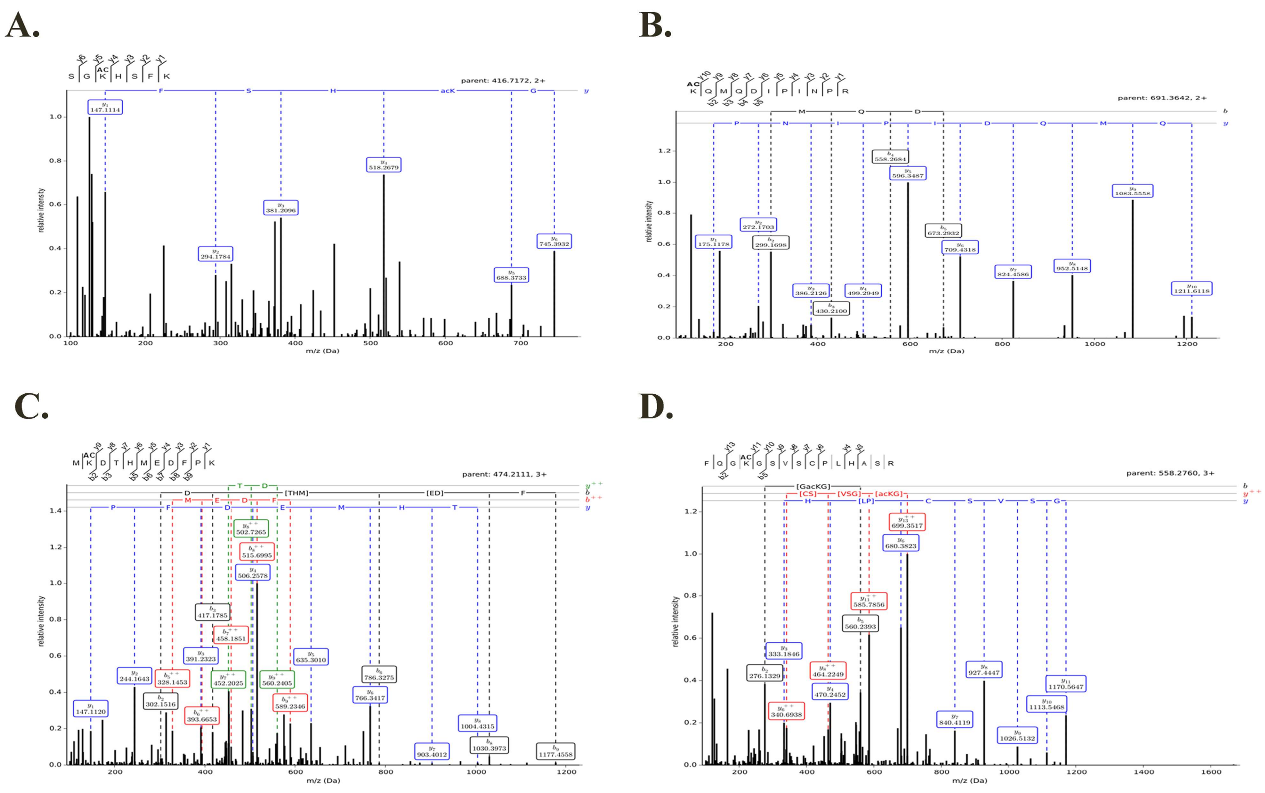

Figure S2. Mass spectrometry analysis of acetylated Polι, recovered from MMS-treated HEK293T cells. Fragmentation spectrum corresponding to respective peptides. Highlighted peaks correspond to b-series ions (blue) and y-series ions (red). Each spectrum of modified peptides was manually curated.

Homo sapiens	VLS**FF**SKKQMQDIP
Pan troglodytes (Chimpanzee)	VLS**FF**SKKQMQDIP
Canis lupus familiaris (Dog)	VLS**FF**STKQMQDSP
Ictidomys tridecemlineatus (Ground squirrel)	VLS**FF**STKQMQDRS
Bos taurus (Bovine)	VLS**FF**STKQKQDSS
Rattus norvegicus (Rat)	VLS**FF**STKQTQAGC
Mus musculus (Mouse)	VLS**FF**STKQMQASR
Meriones unguiculatus (Gerbil)	VLS**FF**STKQMQASS
Myotis lucifugus (Bat)	ILS**FF**SPKQMQDSP
Ornithorhynchus anatinus (Platypus)	VLS**FF**PQKDRQDSD
Equus caballus (Horse)	ILS**FF**SAKQMQDSP
Pelodiscus sinensis (Turtle)	AQP**F**LAQEKMYYSP
Xenopus tropicalis (Frog)	ILN**FF**SRAKAADLP
Danio rerio (Zebrafish)	--S**F**YRPADTED–

Figure S3. Sequence alignment of the Polι RIR peptides from different species. Conserved residues are colored and the essential FF motif highlighted. K550 that in predominantly acetylated in human is marked with yellow.

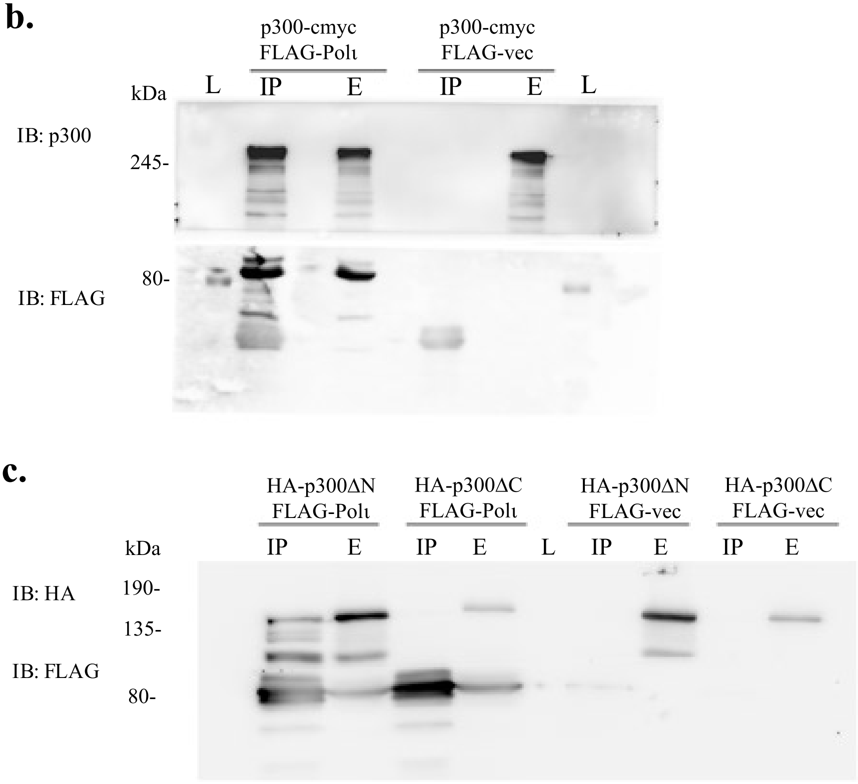

Figure S4. Full-length western blots for Fig 1.

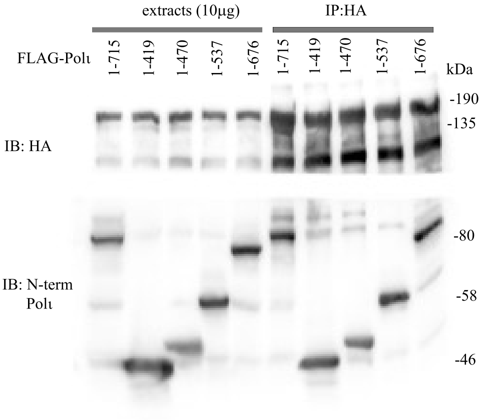

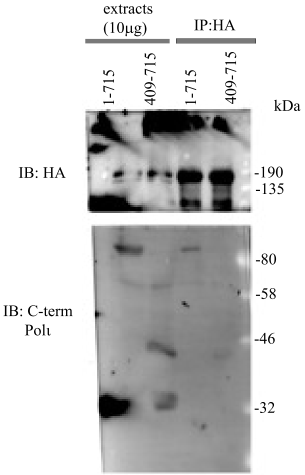

Figure S5. Full-length western blots for Fig 2.

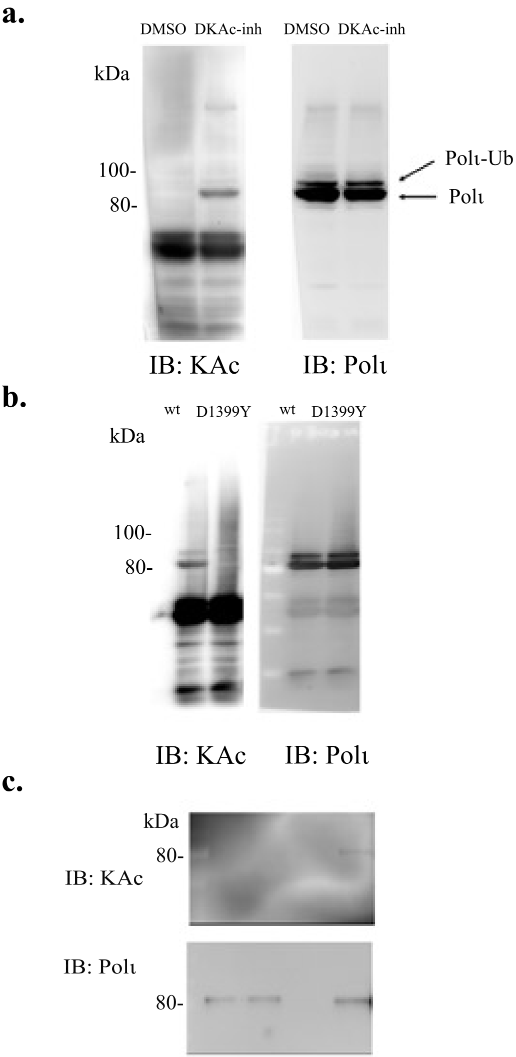

Figure S6. Full-length western blots for Fig 3.

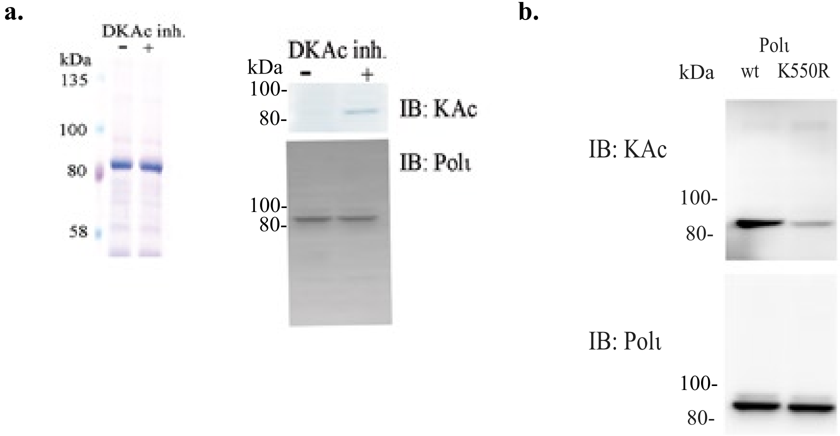

Figure S7. Full-length western blots for Fig 4.

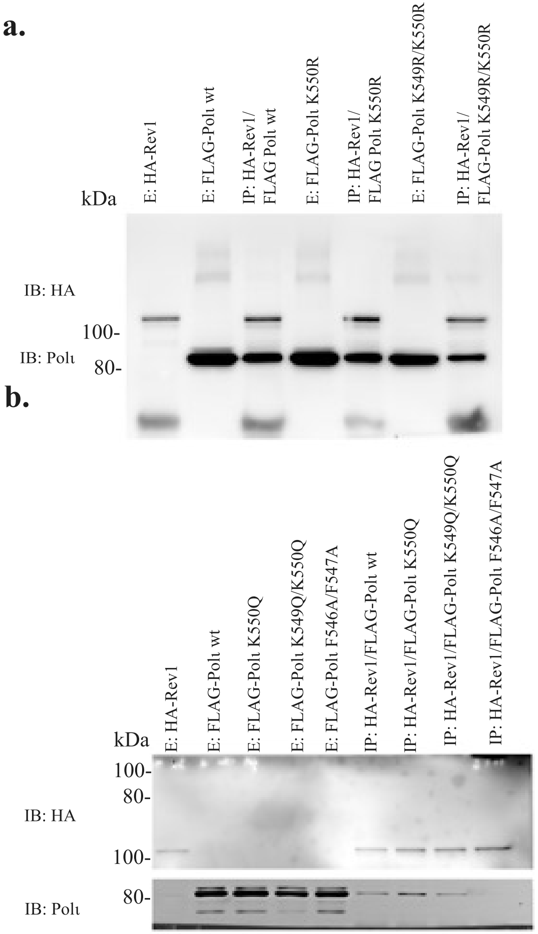

Figure S8. Full-length western blots for Fig 5.

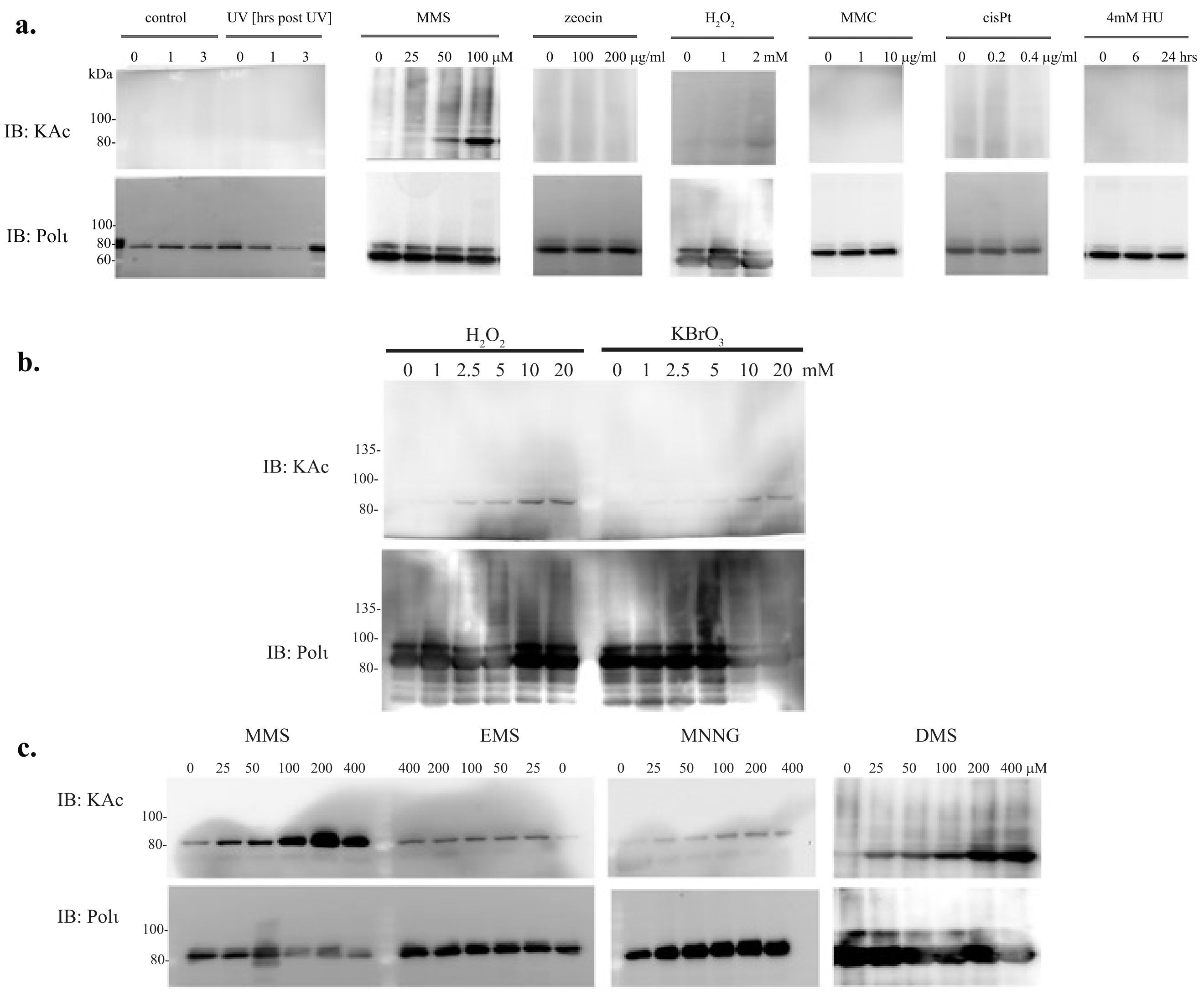

Figure S9. Full-length western blots for Fig 6.

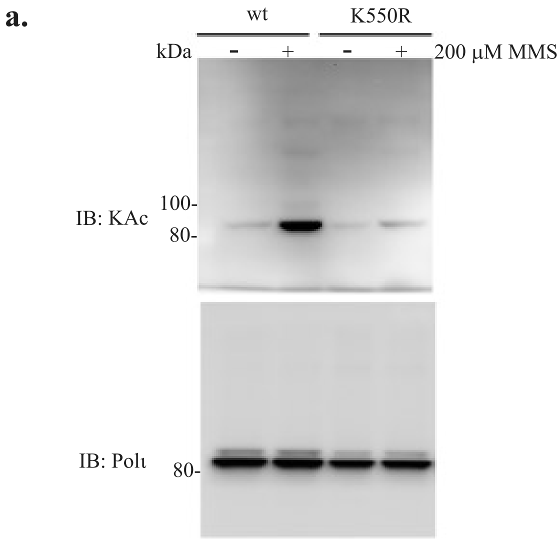

Figure S10. Full-length western blots for Fig 7.

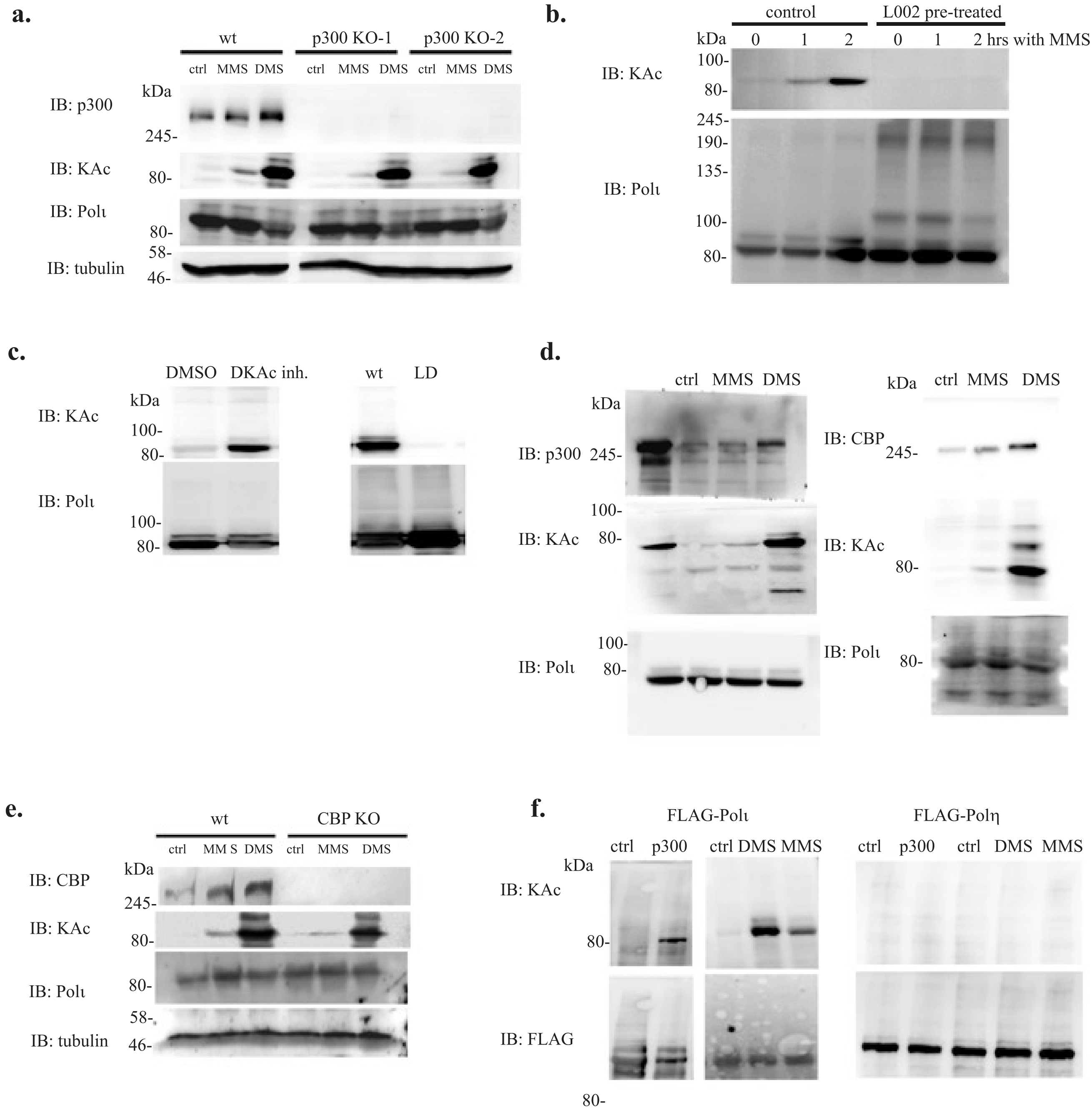

Figure S11. Full-length western blots for Fig 8.
